# Lightweight thermally insulating fired clay bricks enhanced with chitosan-based clay nanocomposites for sustainable construction

**DOI:** 10.1038/s41598-025-11790-5

**Published:** 2025-07-22

**Authors:** M. Abdelhamid Shahat, Wafaa Soliman

**Affiliations:** 1 PV Unit, Solar and Space Research Department, National Research Institute of Astronomy and Geophysics (NRIAG), Helwan, Cairo, Egypt; 2 Geology Department, Faculty of Science, Sohag University, Sohag, Egypt

**Keywords:** Nanocomposite clay bricks, Clay–rGO composites, rGO dopants, Clay shrinkage behaviour, Bricks surface porosity, Thermophysical compositions, Energy science and technology, Materials science

## Abstract

This study explores the enhancement of thermal insulation in fired clay bricks through the incorporation of chitosan (CS) as a biopolymeric dopant. A series of composite samples were prepared with CS concentrations of 0%, 2%, 4%, 6%, and 8%, and their structural, mechanical, and thermophysical qualities were comprehensively investigated. Analytical techniques including X-ray diffraction (XRD), Fourier-transform infrared spectroscopy (FTIR), field emission scanning electron microscopy (FESEM), and thermogravimetric analysis (TGA) were employed to assess compositional and morphological changes. The introduction of CS led to increased XRD peak intensity, indicating improved crystalline organization, while FTIR spectra revealed the presence of CS-associated functional groups. SEM micrographs confirmed the development of a more porous microstructure, and TGA data demonstrated enhanced thermal stability. The CS-modified bricks exhibited an increase in porous topography (33.2–47.9%), a reduction in bulk density (i.e., 1.84–1.29 g/cm^3^), and improved compressive strength (from 0.768 to 1.232 MPa). It is noteworthy that the clay@CS (6%) mix encountered a low thermal diffusivity in addition to the lowest thermal conductivity value (i.e., 0.3418–0.2334 W/mk). The findings show that adding more CS to composite bricks significantly improves their thermal insulation qualities (i.e., 0.314–0.213 mm^2^/S). These outcomes underscore the potential of CS as a sustainable additive for improving the performance of clay-based construction materials, offering promising implications for energy-efficient and environmentally conscious building applications.

**Supplementary Information:**

The online version contains supplementary material available at 10.1038/s41598-025-11790-5.

## Introduction

As a sustainable and green building material, earthen has become more and more prominent in recent years. Globally, houses, archeological sites, and cultural icons are all built using clay. Good mechanical, thermal, and acoustic properties, as well as minimal ecological effects from lowered emissions of greenhouse gases during construction, are among the economic drivers driving this pattern^1^. For this purpose, the possibility of combining clay alongside the bio-polymer CS is beneficial for developing novel and sustainable composites of fired clay bricks^2^. A natural polymer with a distinct cationic character, CS is a harmless polymer generated from chitin, which is present in shellfish^3^. Rather than being a singular polymer with a predetermined structure, it is a member of a family of molecules with diverse monomer distributions, dimensions, and compositions^4^. CS is more desirable from a business standpoint than synthetic cellulose because of its high nitrogen content and inexpensive price. Recently, CS has garnered significant interest due to its attributes and its applications. Whereas, CS finds extensive use in the processing of building, wastewater, medicinal sectors, agriculture, food science, and cosmetics^5^. By simply integrating CS chains into the interlayers of silicate, CS–layered silicate nanocomposites can potentially be produced, improving the mechanical characteristics of silicate. Organophilic nanoscale and natural polymer fillers in clay matrix structures compose the polymer–clay compound category of hybrid substances. An organoclay and a polymer are combined to form a micro-scale composite; the CS serves just as a typical filler. The incorporation and exfoliation are two outstanding nano-scale composites. The intercalation method extends the interlayer gap and produces a well-organized multilayer structure by introducing a small amount of polymer within each layer of clay^6^. CS acts as an organic adhesive to hold the high aspect ratio clay nano-bricks intact due to its charring characteristic. This carbon-based structure, which holds the clay nanoplatelets together, provides a protective barrier that can limit mass and heat transmission within the brick, hence decreasing the degree of thermal energy emission^7^. As constituents of bio-nanocomposites, clay and CS are frequently employed. According to Yao and et al.^8^, the incorporation of CS into clay and the dispersed nature of clay nanosheets in the CS network enhance the mechanical and thermal characteristics of bio-nanocomposites as relative to pristine CS layer. How efficiently CS soaks up on clay is determined by the aqueous solubility of the polymer, which is significantly connected with pH and partly impacted by the type and level of electrolyte used in the mixture^9–11^.

Based on all of this, the use of clay and CS in building materials is an intriguing area of research, combining the benefits of both natural elements to create composites with superior qualities^10^. These composites can provide various advantages over traditional construction materials, such as increased mechanical strength, durability, and environmental sustainability^12^. The inclusion of particles of CS to a clay matrix can greatly increase the tensile strength and toughness of the resultant composite. Clay layers prevent fracture growth; however, the CS network distributes stress more uniformly. The addition of CS particles to clay can also increase flexural strength, making materials more resistant to bending and deformation when loaded^12,13^. Along these lines, CS, as a natural biopolymer, has intrinsic water-resistant qualities; when mixed with clay, which may function as a physical barrier to moisture penetration, the composite material’s resistance to water damage is increased^14^. Incorporating CS into construction materials can suppress the growth of mold, fungus, and bacteria, resulting in not only increased building material longevity but also improved indoor air quality^15^. Improved thermal insulation qualities can assist maintain interior temperature levels and lower energy consumption for heating and cooling thanks to the structure of clay–CS (CCS) composites. In addition, the ability of clay and CS to absorb sound produces better acoustic insulation, which may find utility in soundproofing applications^16^. Since it is derived from the waste of shellfish, it is a renewable and biodegradable resource material with additional advantages for the environment^17^. Consequently, when combined with naturally occurring clay minerals, it lessens their carbon footprint by producing less carbon dioxide, which adds to their allure from an environmental standpoint. Several research teams were looking at improving the mechanical, physical, and thermal properties of earthen building components using a range of strategies, including the use of additives in the material’s manufacturing process^12–20^. Putri and et al.,^16^, used natural clay combined with CS (a gelling agent) and pore pattern in the ceramic structure for thermal insulation activities owing to its high point of melting, chemical inertness, and low thermal conduction. Rasheed and et al.,^13^, employed CS to enhance clay soil stabilization and minimize shrinkage, hence maintaining curling. CS was employed by Ilman and Balkis^15^, to enhance the mechanical features of earthen building supplies, including antibacterial activity, non-toxicity, and biodegradability. Soliman and Shahat^18^, used varying levels of reduced graphene oxide (rGO) nano-filler to optimize the thermophysical performance of clay composite bricks, while Shahat et al.^19^, succeeded in improving the thermophysical performance of fired clay brick nanocomposites by incorporating rGO and TiO_2_ nanofillers. Meanwhile, Trambitski et al.,^12^, used CS to improve the bulk density, shrinkage, compressive strength, hygroscopic behaviour, and resistance to water erosion of clay bricks. As an alternative building material for 3D printing applications, Zavaleta et al.,^14^, investigated the incorporation of natural additives, such as CS, as a soil stabilizer and soil reinforcement to prevent the formation of cracks and boost mechanical and durability attributes. Ferreira et al.,^20^, explored the use of several types of nanomaterials in concrete, coatings, plasters, renders, and thermal insulation—all areas of sustainable building.

Aside from the abundant properties of naturally extracted CS, millions of tons are produced globally each year, which proves economically favorable given the low cost of natural materials. Likewise, clay is the primary material utilized for burnt bricks, along with clay resources may be found all over the world at low extraction and shipping costs. In Egypt, millions of tons of clay are locally accessible and freely spread throughout the country. Delivering new smart substances opens up numerous opportunities to improve thermophysical qualities in clay-based brick usage in the future.

Notwithstanding the known advantages of CS, limited studies have systematically examined its influence on the structural, thermal, and microstructural evolution of clay-based bricks across different doping levels, particularly under high-temperature firing conditions. Therefore, in this study, a series of clay–chitosan composite bricks were fabricated using varying CS contents—pristine clay (CCS0%), clay–chitosan (2%) (CCS2%), clay–chitosan (4%) (CCS4%), clay–chitosan (6%) (CCS6%), and clay–chitosan (8%) (CCS8%)—to investigate their thermophysical, physical, and mechanical properties. Chitosan was selected for its ability to introduce functional groups (i.e., –NH_3_^+^, CH_2_OH and NHCOCH_3_) capable of interacting with clay minerals, thereby tailoring the microstructure and performance of the final bricks. This study provides a comprehensive comparison of these materials, contributing to the broader understanding of biopolymer–clay interactions under thermal treatment relevant to sustainable construction applications.

## Materials and methods

### Materials

**Fig. 1:**
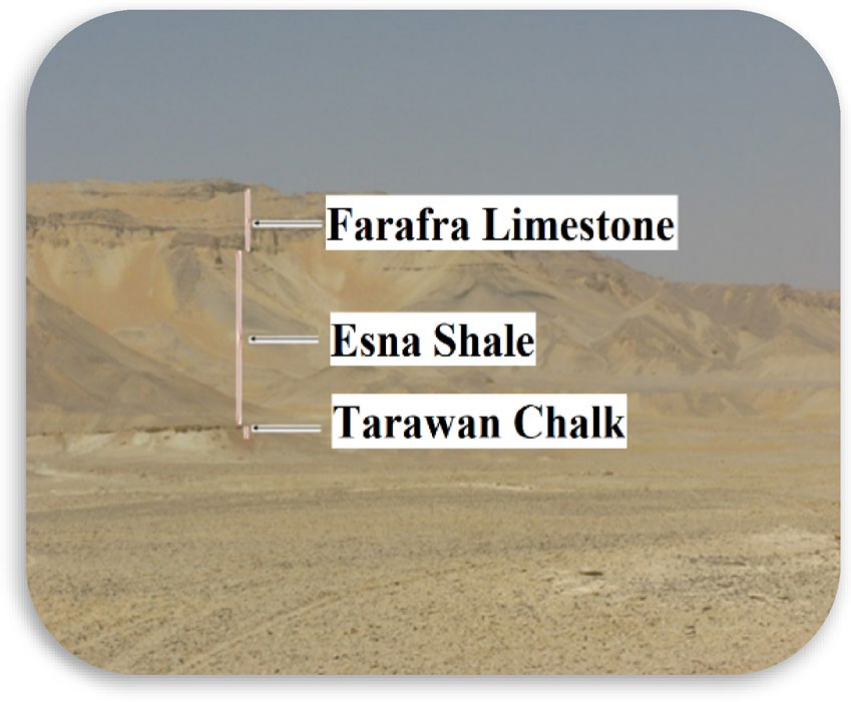
Esna-Egypt formation used in bricks making.

The CS-biopolymer used in this study was purchased from Sigma-Aldrich Corporation. In this study, several concentrations of CS were used for the treatment of the earthen materials. Meanwhile, clay raw material was derived from the field from Esna Formation in Farafra oasis, as seen in Fig. 1. The Esna Formation is a greenish shale and marl with nummulitic intercalations. Its upper part is more calcareous and partly coralline. It is of Paleocene to Eocene age^21^. In the Farafra Oasis, the Esna Formation forms the shale-marl section exposed on the slopes of ElQuss Abu Said Plateau with about 70–100 m thick. It forms the foot of the plateau and underlies the Farafra limestone^22^. The basal part belongs to the Paleocene age while the upper part is of Lower Eocene age^23^. The bulk mineralogy of the clay sample is (Phylosilicates 22%, quartz 4%, calcite 25%, witherite 35%), while the clay fraction mineralogy is (smectite 53%, illie 36%, kaolinite 12%).

#### Chitosan description

CS used in this study is a natural, biodegradable polysaccharide derived from the partial deacetylation of chitin, which is predominantly sourced from crustacean shells. Structurally, CS consists of β-(1 → 4)-linked D-glucosamine and N-acetyl-D-glucosamine units^24^, and its physicochemical properties vary depending on the degree of deacetylation (DD) and molecular weight. The CS employed in this work was purchased from Sigma-Aldrich (USA), with a high molecular weight of approximately 1370 kDa and a reported DD of ≥ 85%. Due to its high viscosity and limited solubility in neutral solvents, it was dissolved in 1% acetic acid where protonation of the –NH_2_ groups promotes full solubilization. The presence of reactive amine (–NH_2_) and hydroxyl (–OH) functional groups facilitates hydrogen bonding and electrostatic interaction with clay minerals, which is beneficial for composite formation. To ensure the structural integrity and purity of the chitosan, a series of characterization tests were performed. FTIR spectroscopy was used to confirm the presence of characteristic bands including the amide I (~ 1650 cm⁻^1^) and amide II (~ 1560 cm⁻^1^) peaks, representing the –C=O stretching and N–H bending vibrations, respectively, as well as broad O–H and N–H stretching bands near 3400 cm⁻^1^. XRD analysis revealed a semi-crystalline structure, with a typical broad peak around 2θ = 20^°^, indicating partial crystallinity often observed in high-DD chitosan. TGA was performed to evaluate thermal stability, showing a two-step degradation profile: initial moisture loss below 120 °C, followed by main polymer decomposition between 250 and 350 °C. These results confirm the thermal behavior of chitosan during the pre-firing phase and its partial decomposition during brick sintering. Furthermore, EDX (energy-dispersive X-ray) analysis was conducted to qualitatively verify the elemental composition of the chitosan sample, particularly the presence of carbon (C), nitrogen (N), and oxygen (O), consistent with its polysaccharide backbone. This elemental profile further supports the high purity and suitability of CS as a biopolymeric additive. Collectively, these characterizations affirm that the chitosan used in this study meets the necessary structural and chemical criteria for effective incorporation into fired clay bricks. Its functional groups and degradation profile contribute to the modification of porosity, enhancement of microstructural bonding, and reduction of bulk density—attributes essential for producing lightweight, thermally insulating construction materials^2,25^.

### Design of composite clay bricks

**Fig. 2:**
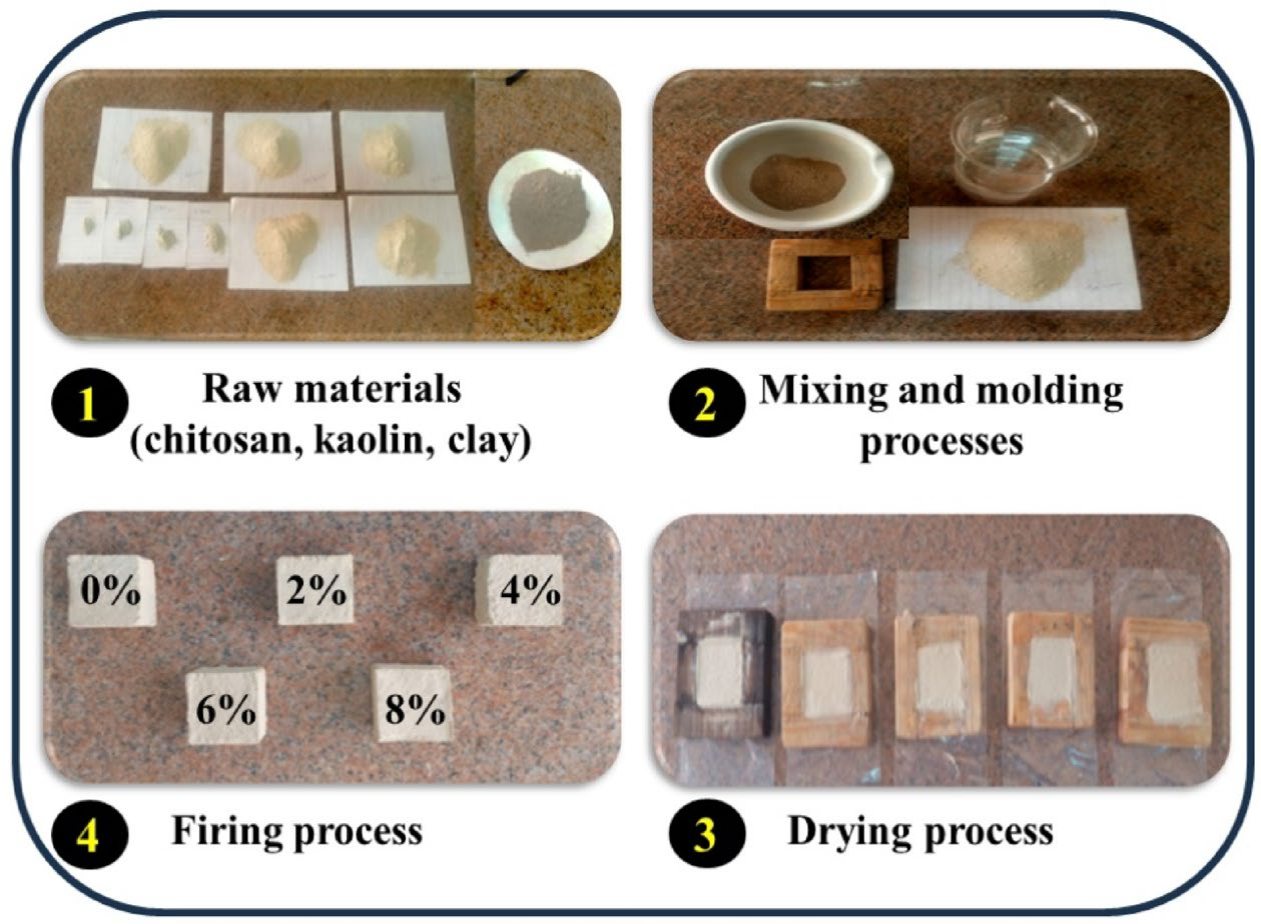
A series of steps for the designing clay bricks.

Clay bricks were formed with different concentrations of CS, and their impact on the different properties of the final composition bricks was investigated. Figure 2 depicts CCS compositions during the molding and firing stages. Each brick comprises up 50 g of solid components mixed with 40 g of tempering water. The first hybrid brick was formed from 50 g (100%) of pure clay, and the second was produced using 98% of clay plus 2% of CS. The third one consists of 96% of clay loaded with 4% of CS. The fourth composite brick consists of 94% of clay loaded with 6% of CS. The fifth composite brick consists of 92% of clay loaded with 8% of CS. The test sections of clay compounds were hand-shaped in a hardwood frame laboratory molding (4 cm × 4 cm × 2 cm). These bricks show well during molding and dried safely with no obvious cracks. To ensure no moisture present, the bricks were naturally dry for 3 days at room temperature and then fired for 4 h at 1100 °C. Firing at 1100 °C gives better vitrification degree in light of the presence of alkali oxides within fired specimens, rendering the brick less susceptible to water absorption and less prone to damage from moisture and weathering.

## Characterizations

Comprehensive characterization techniques were employed to evaluate the structural, morphological, mechanical, and thermal properties of the composite clay bricks. Using X-ray Diffractometry (XRD) with a Cu target (Model D8, Bruker), patterns from 4 to 70° 2θ were discovered, offering insight into the phase development and mineral structure of pure and treated clays. Clay grain structure changes as a result of chemical processing were examined using Fourier transform infrared (FTIR) spectroscopy (Jasco Model 4100, Japan). At room temperature, the IR spectrum generates data with a 4 cm^–1^ resolution in the 4000–400 cm^–1^ area. For a thermal stability test, which was conducted between 23 and 1000 °C, the thermogravimetric analyser (TGA) 1600 SETARAM LABSYS Evo was employed. Within the air surrounds, the cooling and heating speeds were both 10 ˚C/min, with an isothermal accuracy of ± 1 °C. All characterization analyses including TGA, FTIR, and XRD were conducted on the fired clay brick samples after sintering at 1100 °C, in order to assess the thermal stability, structural modifications, and phase composition of the final products. Assessment the clay brick’s size variation during the drying and fire stages at room temperature made it possible to analyze the shrinkage behaviour that resulted from these procedures. Using field emission scanning electron microscopy (FESEM, QUANTA 200 FEG from FEI, Japan) fitted with an energy-dispersive X-ray (EDX) spectrometer, the surface morphology of the hybrid bricks was further investigated in order to determine the surface chemical composition of the constituent parts. Furthermore, bulk density and apparent porosity were measured experimentally using the Archimedes technique. With a force applied centred on the top surface of each sample, the compressive strength was measured using EN 772-1:2011, with each sample’s strength steadily rising until failure. Every material was then evaluated for compressive strength, which was then expressed in MPa. For each test condition, five replicate specimens (n = 5) were prepared and measured to ensure accuracy and reproducibility. The reported compressive strength values represent the average of these measurements. Standard methods were used to test the thermal properties of the burnt clay bricks, such as thermal effusivity, specific heat capacity, thermal conductivity, and thermal diffusivity, in order to assess the insulation effectiveness. Using a Hot Disc Thermal Constants Analyser (Hot Disc TPS 2500 S, Göteborg, Sweden), the measurement techniques were assessed. The transient plane source (TPS) approach, which is widely recognised for its precision and ease of use in examining thermal transport parameters, is utilised by this device. Standard sizes were used to create the samples, which were then analysed at room temperature. For each clay–chitosan formulation, five thermal test specimens were analysed to ensure consistency, and average values was reported. Thermal conductivity was computed utilising the heat flow response over time when the samples came into contact with a sensor. However, to test diffusivity, a heat pulse was supplied to one side of the sample, and the temperature increase on the other side was recorded. By heating the samples and measuring the amount of heat needed to raise their temperature, the specific heat capacity was also evaluated. Using the material’s specific heat capacity, density, and thermal conductivity, thermal effusivity was computed.

## Results and discussion

### Shrinkage behaviour

Clays used to make bricks also have the critical characteristic of shrinking throughout the drying and burning processes. Drying shrinkage is affected by the amount of very fine colloidal particles in the clay, its mineral makeup, and its water content. A ceramic body shrinks as a result of the water content being removed during drying. Therefore, in order to prevent interior flaws, it is crucial to regulate the rate at which water is eliminated throughout the drying process^26^. Drying shrinkage of samples decrease with increasing the CS percent, low adhesion occurs between CS and clay particles, as tabulated in Table 1. Usually, the quality of a brick is considered good if its drying shrinkage is lower than 10%. At different additives concentrations, the calculated values fall within the range of standards^18^. While, firing shrinkage of a brick depends on the volatile contents present, the types of the crystalline phases formed, and the viscosity and surface tension properties. Shrinkage is measured in terms of linear dimensions or volume. Firing shrinkage increases as the CS percent increases because CS is rich in positively charged amino groups interacting strongly with the negatively charged clay surface by direct electrostatic interaction, and as the sintering temperature increased from 900 to 1100 °C, as a result of the interconnection of the clay grains in the specimen body^16^. The lower values of firing shrinkage may be also due to the removal of residual and chemically combined water as well as conversion of additives into ashes which evidently diminish its volume. These chemical reactions during firing along with the rearrangement of grains/particles and orientational ordering in the crystal lattice form a more compact solid texture in comparison to the initial state. Typically, a good brick shrinks by less than 8% ^27,28^.

**Table 1:** Drying and firing shrinkage behaviour of designed clay composite bricks with varying amounts of CS.

Sample	Drying shrinkage (%)	Firing shrinkage (%)
CCS0%	12.7	2.4
CCS2%	10.6	2.6
CCS4%	8.4	2.7
CCS6%	7.9	2.8
CCS8%	8.8	2.8

### XRD analysis

**Fig. 3:**
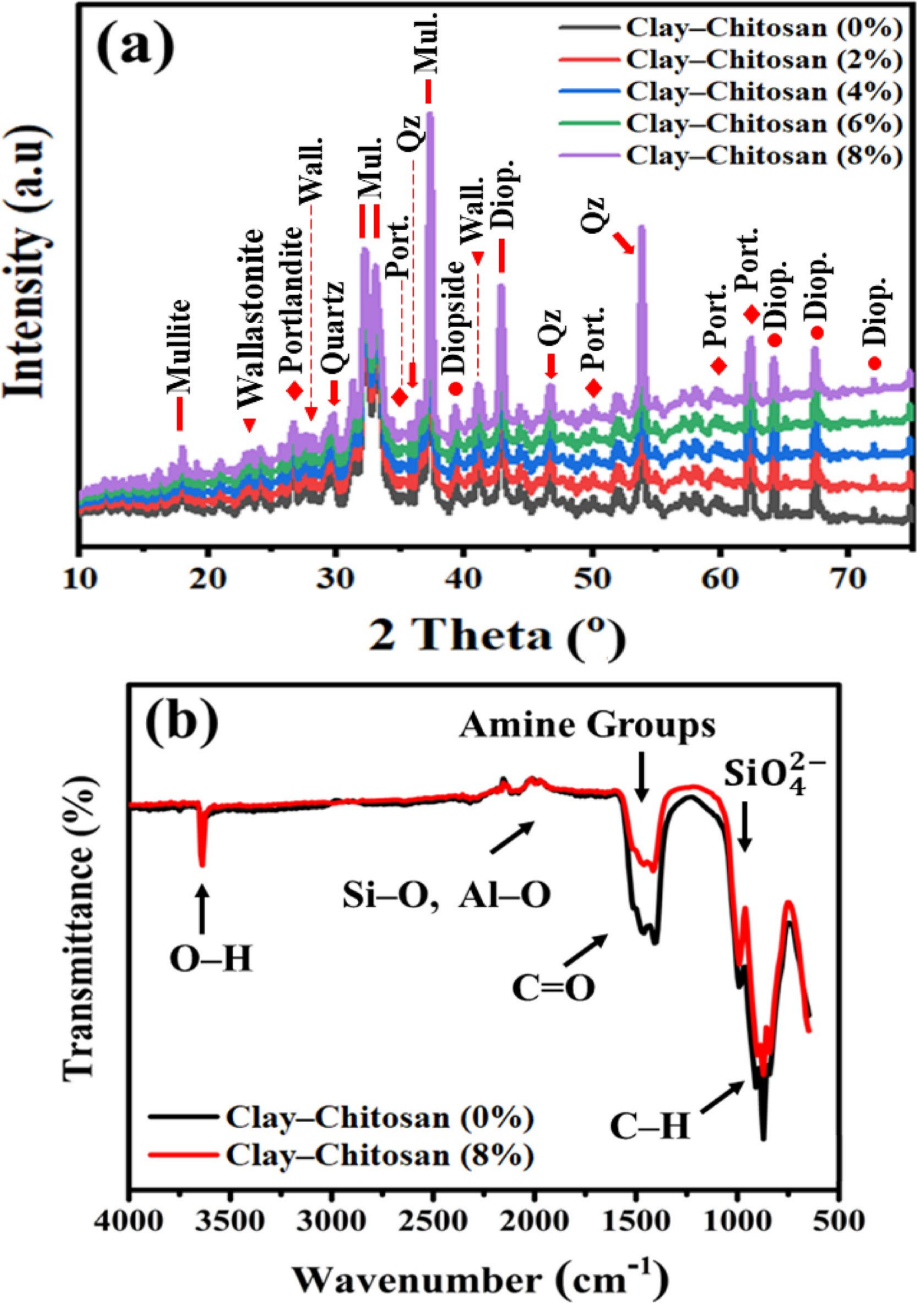
(**a**) X-ray diffraction patterns, (**b**) FTIR spectra of the pure and treated clay using various ratios of CS.

Exfoliation persists as the CS is divided among the layers of clay. As seen in Fig. 3a, there is no CCS phase in the compound because the polymer distribution within the silicate layers of the clay becomes so erratic that they are unwilling to produce an XRD signals^29^. Mullite Al_6_Si_2_O_13_ is formed when kaolinite disintegrates and vanishes (JCPDS card No: 15–776) 2θ = 17°, 26°, 33°, 37°, and 40°^30,31^. Low thermal expansion, low thermal conductivity, and strong mechanical qualities are only a few of the numerous significant characteristics of mullite^32^. The silicate phase (JCPDS 46–1212) (quartz) with 2θ = 28°, 36°, 39°, 40°, 46°, and 50° crystallized is the major mineral phase in these bricks, both with and without additions, that are burnt at 1100 °C^16^. At 2θ = 17°, 27°, 35°, 50°, 55°, 60°, 64°, 77°, and 79°, Portlandite (ICDD #44-1481) could potentially be seen. As the temperature rose, the number of Portlandites increased. Phases containing carbonate and hydroxyl are produced at high temperatures, such as portlandite Ca(OH)_2_, which releases ions of carbonate and hydroxyl (–OH and CO_2_)^33^. Portlandite offers a strong, homogenous structure^34,35^. CaMg-silicates are created when quartz reacts with Ca and Mg ions from carbonate minerals to make diopside (ICDD 11–654) 2θ = 27°, 39°, 43°, 66°, 68°, 73°, and 75°^36^. Diopside gives bricks a high degree of strength^37^, is used to make ceramics with excellent insulation^38,39^, and can withstand chemical corrosion^40^. Decomposed phyllosilicates and carbonates combine to generate wollastonite, which is then converted into the new Ca-silicate mineral CaSiO_3_ (JCPDS card No: 003-0559) Low thermal conductivity material, 2θ = 23°, 26°, 36°, and 43°, is used for producing thermal insulators and foundry lining^41–44^.

### FTIR spectroscopy

In the study of fired clay-based composite bricks, FTIR spectra reveal the chemical changes and bonding modifications that occur when CS is added to the clay matrix at varying concentrations. This analysis is crucial for understanding how different doping levels of CS—2%, 4%, 6%, and 8%—affect the thermophysical properties of the composite bricks, as seen in Fig. 3b. Pristine clay typically exhibits distinct absorption bands corresponding to its primary components: The absorption band near 872 cm^–1^ is attributed to C–H bending plane. The amide I band (stretching vibrations of carbonyl group C=O) appears at 1635 cm^–1^ and associates with the carbonyl groups in the CS’s acetylated units^9^. The peak at 1562 cm^–1^ is related to the N–H stretching vibrations and vibrations of amine groups presents in CS chain. Bands at 1699 and 1631 cm^–1^ are due to the presence of Si–O stretching vibrations, indicative of silicate minerals in the clay, and Al–O groups, respectively. Al–O–Si bending vibrations near 500–600 cm^–1^, representing the aluminosilicate structure^45^.

A broad peak around 1000 cm^–1^ represents the ${\text{SiO}}_{4}^{2-}$ of smectite^17,46^. The presence of SiO_4_ stretching vibrations in the FTIR spectra confirms the existence of silicate structures, predominantly derived from SiO_2_, within the clay matrix. During the sintering process at sufficiently high temperatures, SiO_2_ contributes significantly to the vitrification phase by forming a glassy matrix that promotes densification and bonding among clay particles. This vitrification leads to the reduction of open porosity and enhances the mechanical interlocking between particles, thereby improving the overall strength and density of the fired bricks. Moreover, the incorporation of siliceous materials, such as those found in chitosan-based clay composites, facilitates the formation of strong Si–O–Si linkages that act as a binding phase, further contributing to structural rigidity. These effects have been widely reported in the literature. For instance, Phonphuak et al.^47^, highlighted that the melting and subsequent bonding of silica and shale during firing improve brick strength. Similarly, Leiva et al.^48^, reported increased strength and density due to elevated SiO_2_ and Al_2_O_3_ content during sintering. Additionally, Chindaprasirt et al.^49^, demonstrated the synergistic enhancement in mechanical properties of fired clay bricks through siliceous additives that support vitrification and densification. C–O stretching vibrations near 1020–1070 cm^–1^, corresponding to the glycosidic linkages in the CS polymer. O–H stretching vibrations between 3400 and 3700 cm^–1^ and O–H bending vibrations around 1600–1650 cm^–1^, associated with hydroxyl groups and water molecules absorbed in the clay^29,50,51^.

A sharp band around 2200–2400 cm^–1^ indicates the presence of a C–N or a C–C triple bond^52,53^. 1900 cm^–1^ is reported for carbonates^54,55^. Weak absorption bands at 2250 cm^–1^ and 2350 cm^–1^ indicate Fe–OH and Mg–OH, respectively^56,57^. The 1600 cm⁻^1^ peak can also be assigned to C–O groups^58^. A peak at 900 cm^–1^ is attributed to hydrogen deformations^59^, while a small peak at 700 − 800 cm^–1^ is due to out-of-plane C − H bending^60^. The absorption band at 1153 cm^–1^ corresponds to asymmetric stretching of the C–O–C in CS. The bands at 1066 and 1028 cm^–1^ correspond to C–O stretching in CS^61^, and the peak at 1260 cm^–1^ is assigned to bending vibrations of hydroxyls^62^. At the highest doping level of 8%, the FTIR spectrum exhibits the most essential modifications, suggesting considerable chemical change of the clay matrix. Conversely, there is fluctuation in the N–H and C=O bands, indicating that CS has been extensively incorporated into the clay^63^. The shifts or broadening of the Si–O and Al–O–Si bands indicate that CS has had a significant impact on the clay’s structure. The extensive presence of CS at this level led to the most significant enhancements in thermophysical properties. The high concentration of CS significantly reduces the thermal conductivity of the bricks, making them excellent insulation. Additionally, the modified microstructure results in improved mechanical properties, such as increased resistance to cracking and thermal shock. The FTIR spectrum at this level provides clear evidence of the deep integration of CS into the clay matrix, highlighting the transformative impact of high CS doping levels on the material’s properties.

### Thermogravimetric analysis (TGA)

**Fig. 4:**
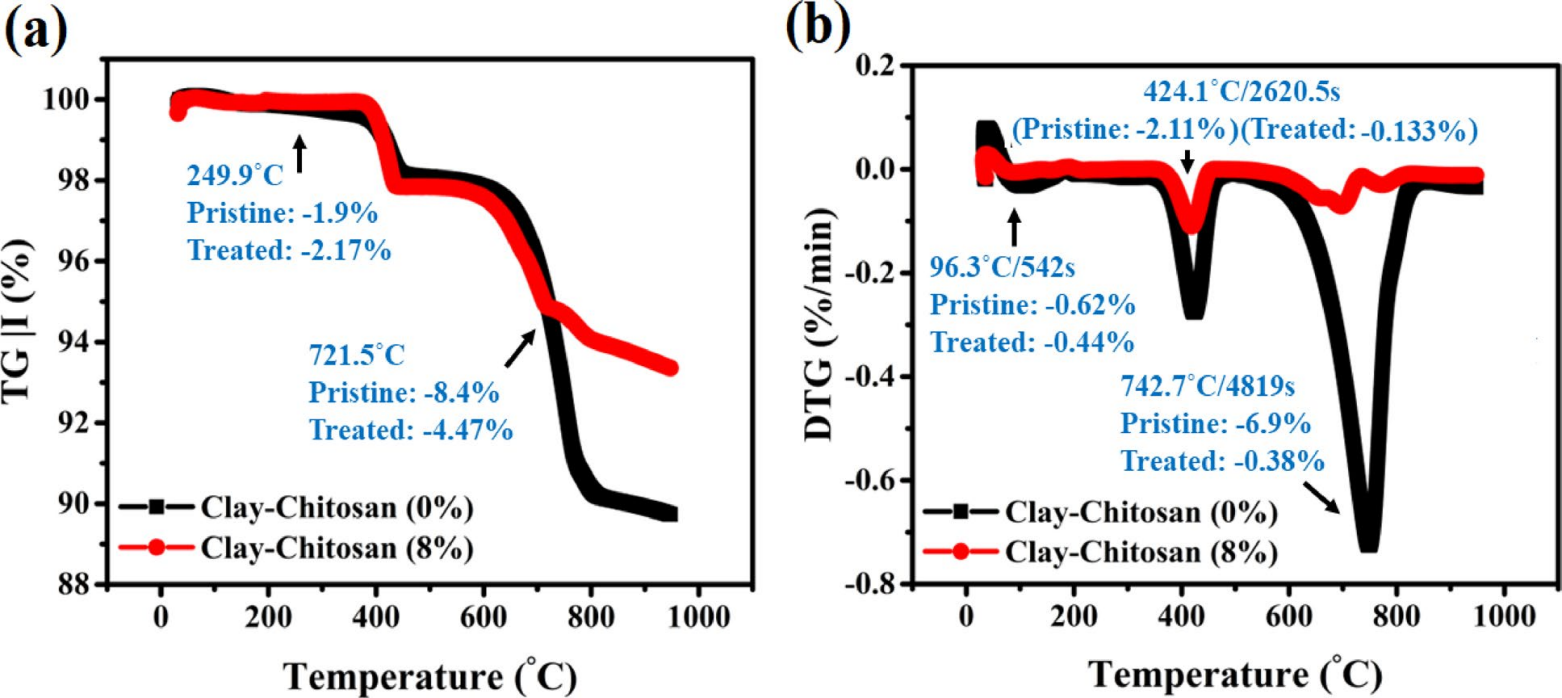
(**a**) TGA, (**b**) DTG curves of the pure and treated clay using various ratios of CS.

According to Fig. 4, the thermal stability of the modified and unmodified clay blends was tested employing TGA and DTG methods for relation to the level of 8% CS employed as a dopant element. Water molecules that were taken within the clay mixtures were the source of the dehydration of clay minerals, depicted in Fig. 4a and b. This covered water that was physically received as well as bound water that was connected to interlayer cations between 25 and 400 °C^64^. As a result, the mass losses at 249.9 °C were –2.17% for CCS8% and –1.9% for pristine clay, respectively. This dehydration phase is consistent with the presence of smectite and kaolinite in the semi-quantitative mineralogical profile of the pristine clay. Smectite, being more expandable, contributes significantly to mass loss in this temperature range due to its high-water content, while kaolinite’s contribution is attributed to surface and interlayer water. With increasing temperature up to 721.5 °C, further mass loss occurred: –8.4% in CCS0% and –4.47% in CCS8%, associated with the breakdown of hydroxyl-containing minerals and organics. Several functional groups, including ${\text{SiO}}_{4}^{2-}$ of smectite^46^ O–H bonds^50^, and carbonyls (C=O)^9^, have arisen as a consequence of the modification induced by the mixing of CS with clay layers. This discovery is supported practically by in-situ XRD data^65^. The DTG peak at 96.3 °C/542 s was notably lower in CCS8%, while more intense in pristine clay, indicating increased thermal stability in the modified material. This can be attributed to the incorporation of CS-derived compounds, including amine groups, carboxylates, and carbonyls, which thermally decompose to release CO, CO_2_, and H_2_O^66,67^. Between 400 and 750 °C, the major mass loss is primarily attributed to kaolinite dehydroxylation, supported by the presence of kaolinite in the pristine clay mineralogy^68^. Additionally, smectite and illite/mica, present in moderate amounts, also contribute to this region through overlapping dehydroxylation processes. At 414.1 °C/2620 s, CCS0% and CCS8% lost –2.11% and –0.133% respectively, indicating reduced volatile content due to CS reinforcement.

In a similar vein the DTG curves of pristine CCS composition reveal a strong peak at 742.7 °C/4819 s with a high level losses of weight mass of –6.9%, suggesting calcite division and illite dehydroxylation^69^. This aligns with the known presence of calcite and illite/mica in the semi-quantitative profile of the pristine clay. In contrast, the CCS8% composite exhibited a DTG peak at the same position but with only –0.38% weight loss, confirming improved thermal resistance due to the stabilizing effect of CS sheets, including carbonyl and quinone moieties. These changes indicate that the CS addition modifies the thermal decomposition pathway by reducing the contribution of heat-labile mineral and organic phases, which is reflected in the overall enhanced thermal stability. From 25 to 1000 °C, the final residue masses were 90% for pristine clay and 99% for CCS8%, suggesting superior high-temperature performance of the doped composite, which is critical for energy-efficient, durable, and thermally resistant fired bricks.

### Morphology

**Fig. 5:**
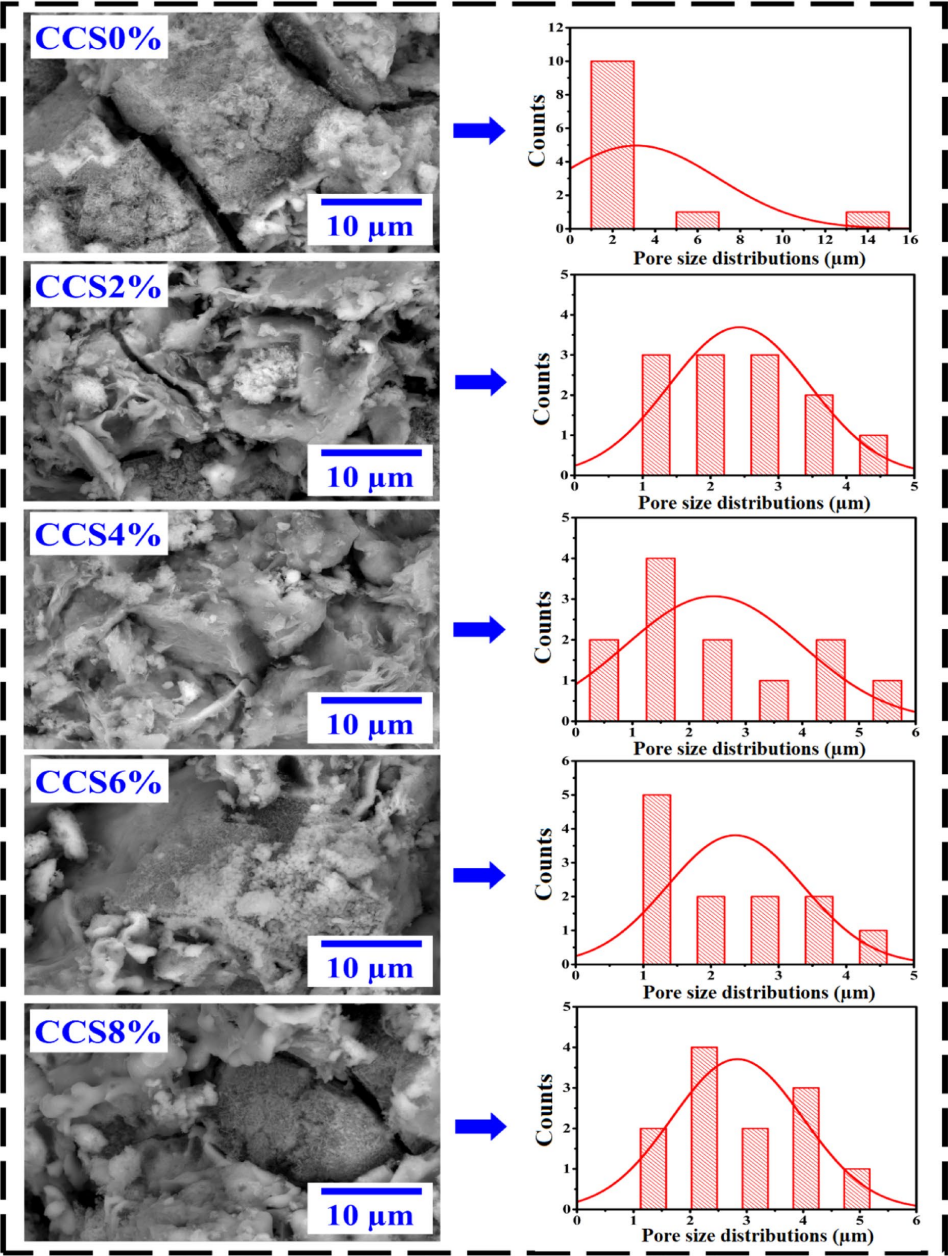
SEM micrographs and statistical histograms of pore size distribution of pristine and modified composite clay using various ratios of CS nanosheets.

Figure 5 presents SEM micrographs and pore size distribution profiles of the modified clay-based CCS composites with varying concentrations of CS dopants, along with the undoped reference sample (CCS0%). The porous morphology of the CCS hybrids varies notably with the doping level, indicating the influence of CS content on pore development. In contrast, the undoped clay (CCS0%) exhibits a uniform, fine-pored surface structure. Additionally, Fig. 5 reveals structural similarities between the CS and clay nanosheets, although their integration patterns differ depending on the dopant concentration. In the meanwhile, a number of crumpled and wrinkly forms were seen in the clay mixtures. This clay-specific characteristic is essential for avoiding unwanted restacking and nanosheet aggregation^70^.

According to these results, there was a homogeneous distribution of CS biopolymers within the clay matrix and the creation of hydrogen bonds between the CS functionalities and the clay medium^71^. At greater CS levels, the alloys’ pattern also grew, displaying an amorphous structure with many linkages, few micropores, and more inner energy^72^. Moreover, statistical estimations were performed of the mean pore diameters of both natural and processed CCS composites. In contrast, the average pore size for CCS0% was calculated to be 3.12 µm; however, by adding CS 2% nanosheets to the clay matrix, this value was reduced to 2.34 µm. Following that, once the CS content rose, the pore size values steadily enhanced and in CCS6% materials, they reached an optimal value of 2.36 µm. The opposite tendency was observed when the amount of CS polymer in the composites was increased; in the CCS8% combination, the estimated size increased marginally to 2.84 µm. The formation of entirely novel intermolecular covalent bonds and hydrogen bonds among clay–H_2_O and CS functional groups—that is, substances containing oxygen, such amine groups, carboxylates, and carbonyls—might account for these discoveries^9,50,73,74^. These linkages cause a consistent dispersion of nanosheets within clay mixtures and affect the pace at which hydration crystals develop^75^. Because of the miscibility of biopolymers, the surface roughness and thermal properties of these composites significantly improved.

### EDX measurements

**Fig. 6:**
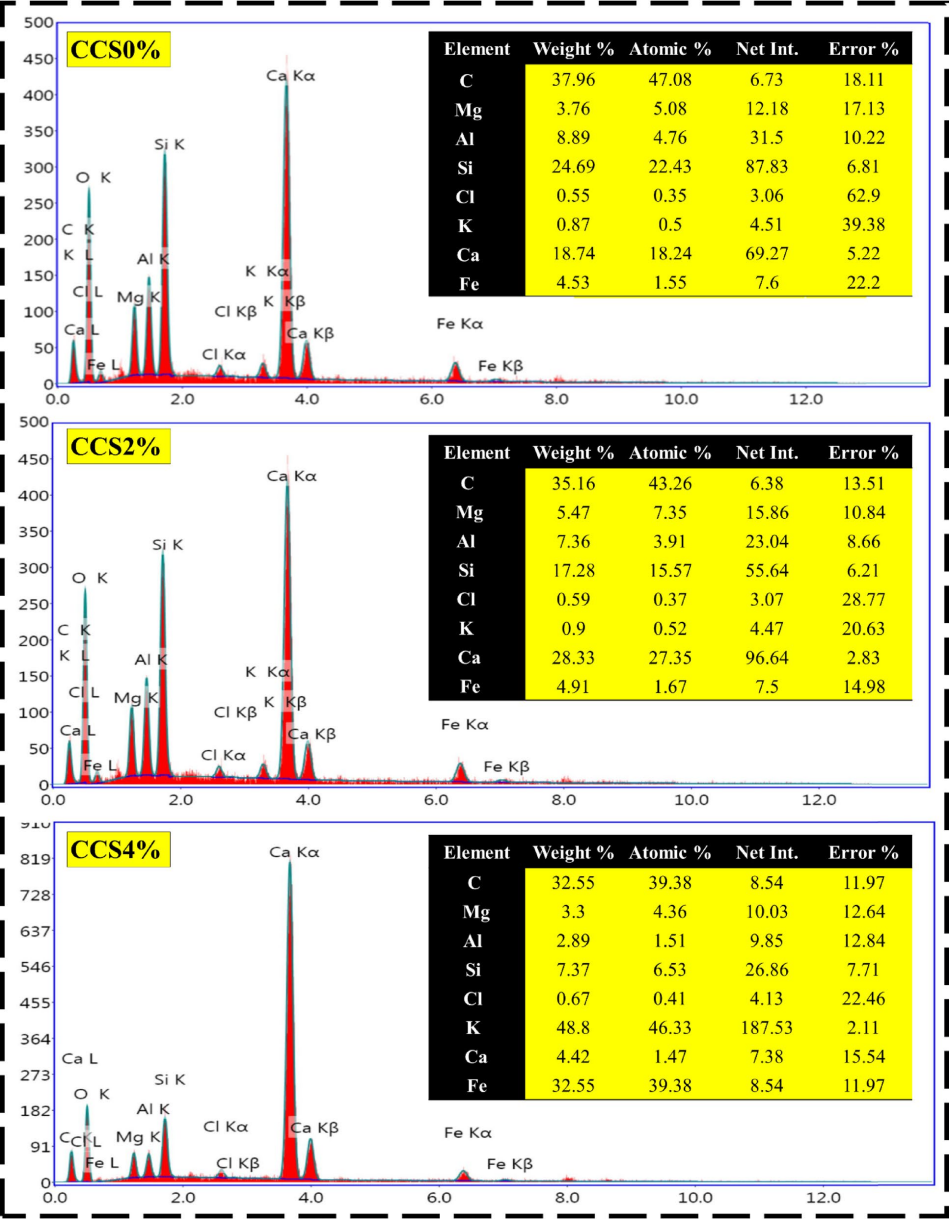

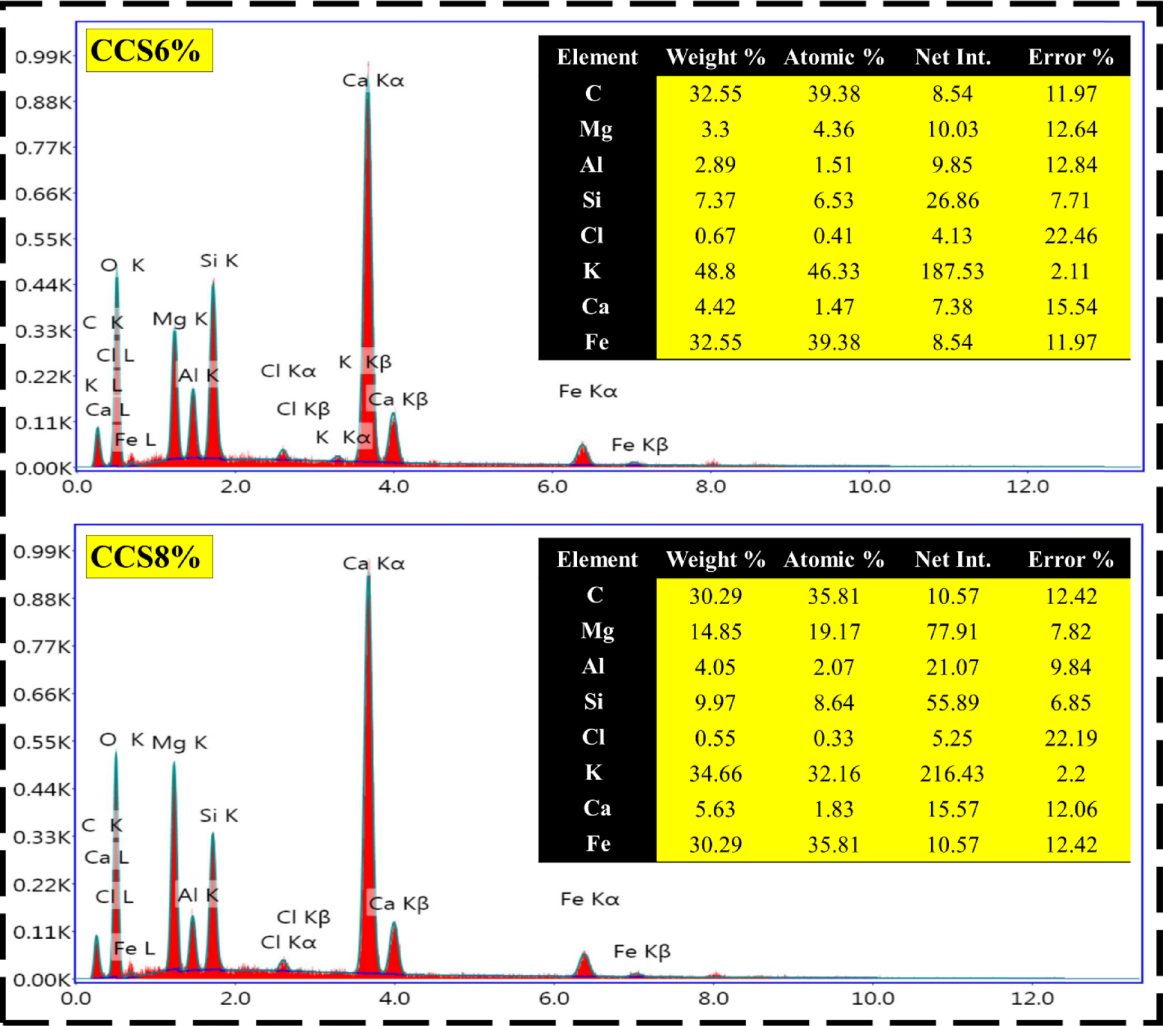
EDX measurements of modified and pure composite clay with different concentrations of CS.

EDX is a valuable analytical tool for investigating the elemental composition and distribution within materials, offering critical insight into how dopants such as CS influence the thermophysical performance of fired clay-based composite bricks^76^. In this study, EDX analysis and mapping were carried out on a series of composite samples containing varying CS doping levels—pristine clay (control), CCS2%, CCS4%, CCS6%, and CCS8%—as illustrated in Fig. 6. More specifically, In the pristine clay sample, EDX reveals a uniform distribution of major elements such as carbon (C), magnesium (Mg), aluminum (Al), silicon (Si), chlorine (Cl), potassium (K), calcium (Ca), and iron (Fe), reflecting the base mineralogical composition of the untreated clay^15,16^. This homogeneity reflects the inherent composition of the clay without any added dopants, serving as a baseline for comparison with the CS-doped samples. At a 2% CS doping level, EDX mapping identifies increased carbon and calcium signals, with localized clusters indicating the initial dispersion of CS into the clay matrix^9^. As doping rises to 4%, the elemental distribution of C, Ca, and nitrogen (N) becomes more prominent and widespread, indicating improved integration of CS. This enhanced dispersion aligns with observed improvements in thermal resistance and microstructural cohesion^10,12^. This suggests better integration of CS into the clay structure, which could enhance the thermophysical properties further. At this doping level, the clay bricks might exhibit improved thermal resistance and possibly some mechanical benefits due to the CS’s ability to bind with the clay particles, creating a more cohesive matrix^13^. As well, with a 6% and 8% CS doping level, EDX mapping typically shows a highly uniform and extensive distribution of the CS-associated elements across the entire clay matrix. The stronger presence of C, N, and Ca reflects deeper penetration and chemical interaction between CS and the clay structure. The improved dispersion indicates a higher level of interaction between the CS and clay, leading to significant enhancements in the thermophysical properties of the bricks^14,15^.

It is important to note that EDX is a surface-sensitive technique, and the incorporation of an organic dopant like chitosan—which is rich in carbon, nitrogen, and oxygen but devoid of inorganic elements like Si, Al, or Mg—can result in apparent fluctuations in elemental proportions across different doping levels. The absence of linear proportionality in the elemental composition (e.g., Mg, Si, Al, Ca) from 0 to 8% CS is therefore scientifically expected. This is due to localized accumulation or heterogeneous surface coverage of CS, as well as its partial decomposition during firing. These phenomena alter the exposed elemental surface detected by EDX and should not be interpreted as inconsistencies in sample formulation, but rather as a reflection of microstructural evolution and organic–inorganic interaction within the fired composite.

In this regard, the high concentration of CS may result in a composite material with excellent thermal insulation properties, due to the increased porosity and reduced thermal conductivity. The mechanical properties might also be affected, with the bricks potentially becoming more resilient to cracking and thermal stress, though the increase in organic content could also lead to some trade-offs, such as a reduction in overall strength or density. Based on the aforementioned findings, the progressive and more uniform distribution of CS, as indicated by the EDX results, directly correlates with the improvements in properties such as thermal insulation, mechanical strength, and thermal shock resistance. The study suggests that higher doping levels of CS, particularly at 6% and 8%, are most effective in achieving a well-integrated composite material with superior thermophysical performance, while also emphasizing the importance of achieving uniform dispersion to maximize these benefits.

### Density and porosity features

As stated in Table 2, the bulk density and apparent porosity of fired clay bricks were greatly affected by the addition of CS polymer as an additional material. As is well known, low-density bricks have a higher thermal insulation potential than standard-weight bricks^77^. By doping the clay with varying levels of CS, a clear trend in density modification was observed. Pristine clay exhibited a bulk density of 1.84 g/cm^3^. However, as CS was introduced, the density progressively decreased, reaching 1.59 g/cm^3^, 1.41 g/cm^3^, and 1.29 g/cm^3^ at doping levels of 2%, 4%, and 6%, respectively. Interestingly, at 8% CS doping, the density slightly increased to 1.44 g/cm^3^. The density of the bricks reduces as the percentage of CS grows, owing to the lower density of the CS biopolymer; however, these values are small and fall within the standard variability for handmade earthen construction bricks^78^. This variation in density is likely due to the structural and microstructural changes induced by the polymer addition. At lower doping levels, the CS likely fills pores and enhances particle bonding, contributing to initial densification^79,80^. However, at higher concentrations, the excess polymer may disrupt the clay matrix, leading to reduced compaction and density. The slight rebound in density at 8% suggests a possible reorganization of the composite structure, potentially due to polymer interactions or phase changes at higher CS levels^81^. These findings demonstrate that CS acts as a density-modifying agent, with optimal levels needed to balance its effects on the clay matrix structure^12^. Notably, CS contributes positively to the production of lightweight bricks. During the firing process, the partial thermal decomposition of CS introduces micro-pores, enhancing porosity and lowering the overall bulk density of the bricks^82–84^. The density reduction effect is particularly desirable in construction materials, offering benefits such as improved handling, reduced structural load, and better thermal insulation^85,86^. Accordingly, CS serves as a friendly pore-forming agent, increasing the number of air pockets and producing lighter and more energy-efficient building blocks.

**Table 2:** Bulk density, apparent porosity and compressive strength features of developed clay composite bricks based on various CS concentrations.

Sample	Bulk density (g/cm^3^)	Apparent porosity (%)	Average pore size (µm)	Compressive strength (MPa)
CCS0%	1.84	33.2	3.12	0.768
CCS2%	1.59	41.7	2.43	0.904
CCS4%	1.41	43.5	2.43	1.085
CCS6%	1.29	47.9	2.36	1.232
CCS8%	1.44	39.8	2.84	1.120

In a similar vein, the addition of CS at varying doping levels—ranging from pristine clay to clay enriched with 2%, 4%, 6%, and 8% CS—demonstrated a clear impact on the microstructure of the bricks. The apparent porosity decreased as the CS content increased, which is consistent with the observed improvement in density. Specifically, the density of the bricks increased by 33.2%, 41.7%, 43.5%, 47.9%, and 39.8% for pristine, 2%, 4%, 6%, and 8% CS doping levels, respectively. This trend indicates that CS enhances the bonding between clay particles, reducing pore size and increasing the compactness of the material ^87^. However, at higher doping levels (8%), a slight decline in density improvement suggests potential saturation in the material’s ability to absorb CS efficiently, likely due to the excessive polymer content that may interfere with proper sintering ^13^. Additionally, thermal processing of clay bricks constructed from basic ingredients including alumina and CS may improve porosity ^16^, according to Salomao and Brandi ^88^. However, at very high concentrations, porosity decreases once more because CS acts as filler within the clay matrix, causing clay particles to agglomerate and drop in the small cavities of the CCS ay blend and obstruct the flow route ^13^. These findings highlight the potential of CS as a polymer additive to improve the structural properties of clay bricks, making them more suitable for applications requiring enhanced durability and reduced porosity^89^.

In broadly, a variety of factors, such as higher porosity and reduced bulk density, may improve both the features and performance of fired clay bricks. The benefits include resistance to moisture, reduced shrinkage and cracking, reduced thermal expansion, lightweight bricks, cost savings, energy-efficient burning, sustainability, and Acoustic and thermal insulation in buildings. Conversely, increasing the number of air pockets within the brick reduces energy consumption and enhances structural insulation by controlling temperature. Earlier studies with a range of dopants, such as fly ash and TiO_2_ NPs, demonstrated a similar bulk density conduct pattern^90–93^. Moreover, it is important to note that the interaction between CS and the clay matrix is largely physical rather than chemical. CS acts as a structural modifier through intercalation or exfoliation within the clay layers, without inducing notable chemical changes during the firing process. This physical interaction contributes to improved pore distribution, reduced density, and enhanced lightweight properties of the bricks^94–96^.

### Influence on mechanical behavior

Table 2 reveals that compressive strength increases with increasing CS along with elevated sintering temperature, indicating that mechanical properties can be improved^16^. This suggests that the CS treatment has a stiffening effect on the treated clay material, which agrees with those who reported that higher material stiffness correlates with higher strength in clay bricks^97^. The addition of the CS-biopolymer to the clay mixture results in a strong attachment between the CS chains and the clay particles, boosting compressive strength. However, increasing volume stability during the air-dried curing process results in less shrinkage and contraction, which is the reason for the improved mechanical properties of clay-CS bricks. Bricks with reduced micro-fissures and improved mechanical qualities were the outcome^46,81,98^. In addition, homogeneity, a highly rigid CCS dispersion, ionic bonding, and a well-organised intercalated structure with robust interfacial adhesion form^99,100^. After CS reaches 8%, strength declines because of clay agglomeration throughout the CS matrix^15,17,100^.

### Thermophysical qualities of fired bricks

**Fig. 7:**
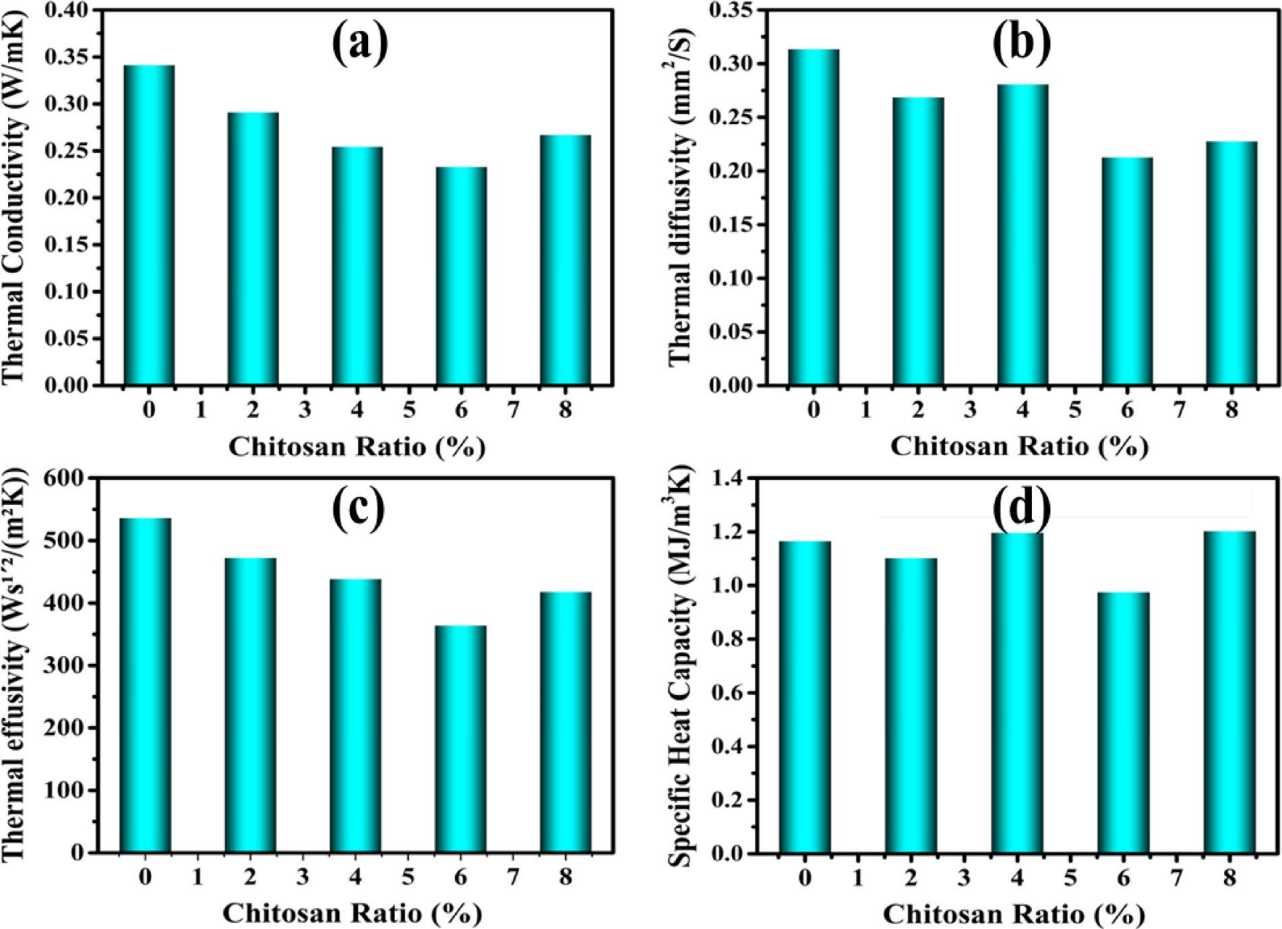
Thermophysical qualities of designed clay composite bricks containing various CS levels.

Doping clay with CS at different levels led to significant improvements in the thermophysical properties of the composite bricks, as shown in Fig. 7. The thermal conductivity, diffusivity, effusivity, and specific heat capacity of pristine and modified clay were determined by its natural composition and microstructure.

#### Thermal conductivity

Figure 7a shows that pristine clay (CCS0%) exhibits a moderate thermal conductivity of 0.3418 W/mK, primarily due to its relatively dense and continuous microstructure, which facilitates heat transfer. With the addition of 2% chitosan (CCS2%), thermal conductivity decreases to 0.2915 W/mK, as the introduction of CS disrupts matrix uniformity and introduces microvoids that act as barriers to heat flow^98^. At higher doping levels—4%, 6%, and 8% CS—this trend continues, with further reductions in thermal conductivity due to increased porosity and more pronounced disruption of thermal conduction pathways. The optimal thermal insulation is achieved at 6% CS (CCS6%), where thermal conductivity reaches a minimum of 0.2334 W/mK. This reduction is attributed to the formation of more organic domains and nano-voids, which inhibit phonon propagation and thus reduce heat conduction^101^. Although CS undergoes thermal decomposition during firing at 1100 °C, its influence on thermal behavior remains significant. During the pre-firing stage, CS acts as a pore-forming agent, improving particle dispersion and microstructural packing. Upon decomposition, CS leaves behind carbonaceous residues and a porous microstructure, which act as phonon scattering centers, interrupting the heat flow and suppressing thermal conductivity. Hence, CS’s influence is primarily physical and structural rather than chemical post-firing. The residual structure left by CS enhances thermal insulation through interface-induced phonon scattering and microstructural disorder. Furthermore, pre-firing interactions between CS chains and silicate layers may lead to nanoscale intercalated or exfoliated structures, adding additional barriers to phonon transport^98,102^. The cationic nature of CS also facilitates encapsulation of clay particles, altering interfacial dynamics and reducing thermal transport efficiency^103^.

#### Thermal diffusivity and effusivity

CCS0% exhibits a moderate thermal diffusivity, reflecting a balanced capacity to conduct and store heat, though not optimized for insulation purposes. Upon introducing 2% CS (CCS2%), the thermal diffusivity decreases slightly due to increased internal resistance caused by microvoid formation and reduced matrix uniformity. As seen in Fig. 7b, the heat spreads more slowly through the material, indicating an improvement in thermal resistance. Simultaneously, thermal effusivity—the ability to exchange heat with surroundings—also drops (Fig. 7c), as CS reduces material density and alters thermal interaction mechanisms. At 4% CS (CCS4%), an unexpected increase in thermal diffusivity is observed despite a lower thermal conductivity. This behavior can be attributed to a combination of moderate porosity and reduced bulk density, which may lead to a decrease in heat capacity^13^. As a result, the material responds more quickly to temperature changes, increasing diffusivity even though it conducts less heat overall. Similar effects have been reported in porous or lightweight composites where a balance between conductivity, density, and specific heat governs thermal diffusion rates. As CS content increases further to 6% (CCS6%), the diffusivity drops significantly to 0.213 mm^2^/S, indicating a strong resistance to heat propagation due to increased microvoid formation and disruption of thermal pathways^104^. This is associated with enhanced insulation performance. However, at 8% CS (CCS8%), a slight increase in thermal diffusivity is observed. This may be due to carbonaceous residues forming a more interconnected structure during firing, allowing localized pathways for heat to travel more efficiently. Additionally, CS aggregation at high concentrations may lead to partial densification and clustering effects, slightly reducing internal resistance and contributing to this marginal rise in diffusivity.

In parallel, thermal effusivity consistently decreases with increasing CS content. The CCS6% sample exhibits the lowest effusivity value (364.64 Ws^1^′^2^/m^2^K), indicating a reduced capacity for exchanging heat with the environment and improved internal thermal stability. This behavior is favorable for insulating materials that must buffer rapid external temperature changes. The final formulation at 8% CS still maintains low effusivity, though slightly higher than CCS6%, consistent with the marginally increased density and connectivity due to carbon residues^105^. Overall, these results demonstrate that CS incorporation alters the thermal performance of fired clay bricks through microstructural and physical mechanisms rather than chemical one’s post-firing^86^. The observed thermal diffusivity and effusivity behaviors reflect a complex interplay between porosity, specific heat, residual carbon structures, and particle aggregation—supporting CS’s potential as a thermal insulation enhancer in fired ceramics.

#### Specific heat capacity

The specific heat capacity, which reflects the amount of heat required to change the temperature of the material, is relatively stable, indicating a standard ability to absorb and store heat. The specific heat capacity was slightly increased, as CS introduced new chemical bonds (CCS2%) and structures that require more energy to alter their temperature, as displayed in Fig. 7d. This tendency also continued to rise slightly in CCS4% brick due to the increased complexity of the material’s internal structure, which requires more energy for temperature changes. While, it was optimized in CCS6% matrix (0.976 MJ/m^3^K), reflecting the material’s increased ability to store heat. This is beneficial in applications where maintaining a stable temperature over time is important. The specific heat capacity, which reflects the amount of heat required to change the temperature of the material, is relatively stable, indicating a standard ability to absorb and store heat. The specific heat capacity was slightly increased in CCS2%, as CS introduced new chemical bonds and structures that require more energy to alter their temperature, as displayed in Fig. 7d. This tendency also continued to rise slightly in CCS4% brick due to the increased complexity of the material’s internal structure, which requires more energy for temperature changes. While, it was optimized in CCS6% matrix (0.976 MJ/m^3^K), reflecting the material’s increased ability to store heat^106^. This is beneficial in applications where maintaining a stable temperature over time is important. The following explanations could be given for these observations:

- The first is that when silica and CS combine, their structure retains a high number of nanopores on its surface, which lowers the amount of heat transfer between molecules. A large number of pores prevents heat conduction and slows down the rate of heat transfer^107^.
- The second reason is that the combination of clay and CS causes a laminar stacking of sheets with space between them, which inhibits the transfer of heat^108,109^.
- The third explanation is that heat transfer is reduced when clay and CS sheets aggregate. Because it takes longer for an insulator to heat up after absorbing more heat, they typically have a high specific heat capacity^110^.

Table 3 presents an overview of the mechanical assessment and shrinkage behaviour of various clay-CS composites utilised in the production of burnt clay bricks, as documented in the literature. It also contrasts the performance quality of the designed bricks with those of bricks that are currently in use and were made with varying amounts of modified CS. Certain clay bricks performed worse than the current CCS6%'s compressive strength (1.232 MPa), as Table 3 demonstrates. The substantially greater surface area of the CS-modified clay bricks, which may depend on an elevated rate of absorbing water molecules, is the likely clarification for this improvement in clay system features^111,112^.

**Table 3:** A comparison of the mechanical assessment and shrinkage behaviour of clay-CS composites used in the production of burnt clay bricks with their current study.

Designed Bricks	Compressive strength (MPa)	Shrinkage (%)	References
Clay–Chitosan (6%)	1.232	Drying (12.7–1.9) Firing (2.4–2.8)	Current Work
Clay–Chitosan	18–42%		^12^
Soil – Chitosan		12.57–14.18	^13^
Clay–Chitosan	1.90		^14^
Dry soil – Chitosan	27%		^15^
Clay–Chitosan		4.01–5.74	^16^
Soil – Chitosan	0.200		^17^
Clay–rGO (4%)		Drying (16.1), Firing (5.2)	^18^
Clay–rGO–TiO_2_		Drying (11.3), Firing (6)	^19^
Clay–Eggshell waste (10%)	0.05	Drying (12.5), Firing (1.96)	^22^
Earthen materials –Chitosan	85%		^81^
Clay–Chitosan	65.67		^98^
MgO–TiO_2_ (10%)		0.2–1.1	^113^
Sandy soil – Chitosan	0.300		^114^
Soil aggregates – Chitosan	0.200		^115^

## Conclusion

The incorporation of CS into fired clay-based composite bricks has demonstrated a notable impact on the thermophysical, structural, and mechanical performance of the materials. Through a systematic variation of CS doping levels (0%, 2%, 4%, 6%, and 8%), the study confirmed that increasing CS content results in enhanced thermal insulation properties—evidenced by a marked reduction in thermal conductivity (i.e., 0.3418–0.2334 W/mk), thermal diffusivity (down to 0.213 mm^2^/s), and thermal effusivity. Among the tested composites, the clay–CS (6%) blend emerged as the most effective, exhibiting the lowest thermal conductivity and diffusivity, which makes it particularly suitable for applications requiring high thermal resistance. This performance is attributed to the structural reorganization within the clay matrix, including increased porosity, lower bulk density, and chemical modifications, as confirmed by FTIR, XRD, and SEM analyses. The FTIR spectra revealed new bonding interactions between CS functional groups and the clay matrix, including C=O, N–H, and Si–O–Al shifts, which indicate successful chemical integration. XRD results showed increased peak intensity, suggesting enhanced crystallinity and structural ordering due to CS doping. Furthermore, SEM images illustrated a more heterogeneous and porous microstructure, contributing to the observed thermal behavior. Mechanically, the addition of CS also led to improved compressive strength (up to 1.232 MPa) and increased apparent porosity (from 33.2 to 47.9%), while the shrinkage behavior varied with CS content, influenced by water retention and organic burnout during firing. Overall, the study highlights CS as a promising sustainable additive for enhancing the energy efficiency of clay-based construction materials. The multifunctional benefits—reduced thermal conductivity, improved porosity, and better mechanical integrity—suggest that clay–CS composites, particularly at higher CS contents, are viable for use in eco-friendly, thermally insulating building materials. These findings open opportunities for integrating biopolymer-based dopants in traditional ceramics to meet modern standards of sustainability and energy conservation in construction.

## Electronic supplementary material

Below is the link to the electronic supplementary material.

Supplementary Material 1

Supplementary Material 2

## Declarations

### Data availability

The datasets generated and/or analysed during the current study are available in the Crystallography Open Database (COD) repository, under accession number [COD ID: 3000607] at https://www.crystallography.net/cod/3000607.html.
